# A Protocol for Evaluating Contextual Design Principles

**DOI:** 10.3390/bs4040448

**Published:** 2014-11-07

**Authors:** Arthur Stamps

**Affiliations:** Institute of Environmental Quality, 290 Rutledge Street, San Francisco, CA 9410-5344, USA; E-Mail: InstituteOfEnvironmentalQuality@comcast.net; Tel.: +1-415-282-4005

**Keywords:** contextual design, Cochran Review, evidence-based urban planning

## Abstract

This paper explains how scientific data can be incorporated into urban design decisions, such as evaluating contextual design principles. The recommended protocols are based on the Cochrane Reviews that have been widely used in medical research. The major concepts of a Cochrane Review are explained, as well as the underlying mathematics. The underlying math is meta-analysis. Data are reported for three applications and seven contextual design policies. It is suggested that use of the Cochrane protocols will be of great assistance to planners by providing scientific data that can be used to evaluate the efficacies of contextual design policies prior to implementing those policies.

## 1. Introduction

Contextual design—the concept that new projects should fit into their contexts—has been and is a major planning principle. More than 90% of the cities in the United States, as well as cities in many other countries, regulate architectural aesthetics [1,2,3,4,5,6,7]. The importance of contextual fit stems from the fact that it is a major evaluation criterion in design review. Lightner [2] found that 77% of U.S. planning departments evaluated new buildings using the general criterion of how well the building would fit into its context. In addition, contextual design is relevant to other planning goals, such as facilitating tourism [8], attracting a highly-skilled workforce [9], coping with the increased density, which is integral to the design of sustainable cities [10], and promoting health by increasing movement by foot rather than by car [11].

Given the importance of contextual design, it would be useful for planners to know how well various contextual design principles actually work. In this context, the language “work” is taken to mean that, if applied, a contextual design principle would enhance visual appeal. Typically, whether an urban design principle does or does not work is made using experience, deliberation, rhetoric and authority, and differences are attributable to demographic distinctions. Examples are given in Gans’ classic *Popular culture and high culture* [12] and personified by Howard Roark and Ellsworth Toohey in *The Fountainhead* [13]. Another option is to focus on how the information needed to validate an urban design principle is obtained rather than who does the evaluation. A study of environmental aesthetics covering 277 studies, 41,000 participants, 12,000 stimuli, as well as evaluation methods of a citizen review, planning staff evaluations as given in urban design codes and decisions of blue-ribbon committees by authorities, suggested that scientific methods work much better than traditional methods [14]. For example, the correlation between public responses to as-built projects and decisions between blue-ribbon committees, citizen review boards or houses that did or did not conform to contextual design regulations was, on the whole, *r* = 0.09 [14] (p. 283). On the other hand, the correspondence between preconstruction scientific experiments and post-construction evaluations was *r* = 0.86 [14] (p. 282). These results suggest that it would be worthwhile to integrate scientific evidence into the planning of contextual design principles. As it happened, this same question was raised, and answered, in the medical community in 1972. The name of the medical solution is the Cochrane method. This article attempts to demonstrate how the Cochrane method can be applied to the issue of evaluating contextual design principles.

### 1.1. The Cochrane Method

The proposed solution for testing urban design principles is based on two works: *Effectiveness & efficiency: random reflections on health services*, by Archie Cochrane [15], and *Primary, secondary, and meta-analysis of research*, by Gene Glass [16]. The problem that Cochrane addressed was “The main job of medical administrators is to make choices between alternatives. To enable them to make the correct choices, they must have accurate comparable data about the benefit and cost of the alternatives.” [15] (p. 25). One connection between this issue and the issue of contextual design is clear: they are the same issue, but are applied in different contexts. A typical medical application would be to decide which of two drugs cures the most patients; a typical urban design application would be to decide which of two design guidelines has the stronger effect on a criterion, such as visual appeal.

Cochrane’s solution has two parts. The first part is collecting original data. For medicine, the sources are “randomized controlled trials”, or RCT [15] (p. 2). For urban design guidelines, the sources will be “randomized controlled experiments”. If only one alternative is being investigated, then it is compared to a random sample (in drugs, a placebo; in urban design, a random sample of a neighborhood), and the resulting conclusion is whether the alternative has an effect that is better, the same as or worse than the random sample. For instance, a design guideline on heights could be validated by conducting an experiment in which some images showed existing blocks of buildings with equal heights and other images showing the block with one building that was taller than the rest. For a two-alternative decision, such as adding street trees or prohibiting cars, the experimental validation would involve how strongly each option changed a criterion, such as visual appeal, and then using that data as the benefit in the cost-benefit analysis required for quality control. That is the promise, which leads, inevitably, to the question of how such experimental designs can actually be done.

#### 1.1.1. An Experimental Protocol

A very simple, yet highly efficient, protocol for obtaining data on effects, such as visual impacts, requires the creation of images showing the alternatives (before and after or proposed and a random sample of an existing one). Then, semantic differential ratings (such as (1) not appealing to (8) appealing) are obtained. Third, appropriate statistical tests are done to ascertain which, if any, alternatives are better or worse than other alternatives. The most common statistical test is a contrast between alternatives. Details on how to calculate contrasts are given in Rosenthal and Rubin [17]. Working examples are given in Winer, Brown and Michels [18]. Computer implementations are given in Tabachnick and Fidell [19].

**Figure 1:**
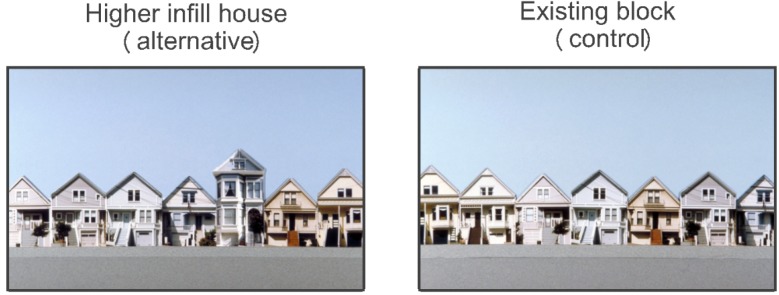
These two images show a difference due to the presence or absence of one environmental feature: an infill house that would be higher than the existing houses.

For instance, Figure 1 shows two images that could be used to test whether the visual appeal of a block would be diminished by allowing a new infill building that would be taller than the existing buildings. The existing buildings would be the control group, and the new infill house would be the urban design alternative. When the experiment was done [20], the mean rating on the criterion of visual appeal was *M* = 5.51 for the existing block and *M* = 4.94 for the blocks with one taller infill building. Since 4.94 is less than 5.51, the presence of a larger infill building decreased the visual appeal of the block. Numerically, the difference in means was 5.51–4.94 = −0.57. Statistically, this difference achieved something called a “*p*-level” (For readers with statistical backgrounds, the “*p*-level” is actually the probability of an alpha error or the probability of reporting a false positive. The term “*p*-level” is used here, because behavioral scientists are more likely to be familiar with it than with the concept of alpha errors). The “*p*-level” indicates the probability that a finding could be due to chance. The conventional interpretation of a “*p*-level” is to accept a claim if *p* < 0.05. Finally, in order to make the findings compatible with findings from other experiments, the difference between means is converted into another measure: a correlation. The symbol for a correlation is *r*. No content is lost in this conversion. It is like measuring temperature in Celsius rather than Fahrenheit. Either measure will do equally well. The advantages of using *r* are explained in Rosenthal and Rosnow [21]. For the contrast between the existing block and the alternative, the contrast, reported as a correlation, was *r* = −0.27.

#### 1.1.2. Validation of the Proposed Protocol

Of course, the utility of this simple protocol for urban designers depends on the validity of the protocol. There are numerous possible difficulties. One possible objection is that environments cannot be represented by static color images [22]. Because the issue of simulation validity is so important, a considerable amount of research has been devoted to the Beaux Arts hypothesis. The basic paradigm for testing simulation validity is to obtain the same responses in the field, where people can look, turn or move around at will and then obtain responses using simulated environments. Over the past 30 years, this paradigm has been applied to many media. The media includes dynamic virtual reality models. Responses include ratings of pleasure, naturalness, familiarity, order, inertia, arousal, threat, disliking, liking of the environment, being nice area to walk through, a good area to live in, appreciation of the area, visual appeal, evaluation, ambience, arousal, privacy, security, pleasant, interest, comfortable, excited, playful, water, built, water flow, sun, sound, strolling, resting, talking, observing, preference and spaciousness.

The current data cover 967 environments and 6323 participants. Overall, responses obtained from static color images agree very strongly (*r* = 0.86) with responses obtained in the field [23]. From an evidence-based point of view, the topic of simulation validity has moved beyond the general question of whether simulations are valid at all for providing guidance regarding which refinements of simulations are most effective in different contexts. For example, measuring visual impacts along streets can be done with two images, while visual impacts in areas, such as open landscapes, will require four images [24].

Other possible difficulties might be the use of semantic differential scaling, how to represent affective responses, possible demographic effects, whether results from single experiments can, in fact, be replicated and whether the proposed protocol has worked in real-world applications. Again, there are considerable data on these issues. On the issue of semantic differential scaling, a review of studies from many researchers, covering 1,150 stimuli, indicated that many common methods of scaling (ratings, rank orders, Q sorts, physically placing stimuli on a table, raw score, comparative judgment, true score and signal detection theory) generated findings that were virtually identical (*r* = 0.99) [14] (pp. 98–101). For a model on how to represent affective responses, a review of work by many researchers generated data covering 7,168 participants, 1,768 environments and 23 possible measures. The overall finding was that affect can be measured in terms of three basic dimensions: pleasure, arousal and dominance. For the current article, the only relevant dimension is pleasure, which can be expressed as dislike/like, not appealing/appealing, ugly/beautiful or any synonyms thereof. For demographic effects, the current data, also obtained from the work of many researchers, covers 19,000 participants from 23 countries, 3,821 environments and 12 demographic groups. There was a very high consensus on the criterion of visual appeal for all demographic groups, except for adults and children ages 12 or younger, general population and designers for *avant-garde* projects and special interest groups, such as industry lobbyists or neighborhood groups. Furthermore, data are available regarding the reproducibility of the simple protocols recommended in this article. The result is that these protocols have been highly reproducible (*r* = 0.90). References to the data that support these validation claims are given in Stamps [25]. Real-world case histories of how the proposed protocols have been used include an *avant-garde* building, a determinant building envelope, an architectural competition, a study in scale and character, citizen participation, restoration after an urban forest fire and the design of a unique house [14] (pp. 185–271). Overall, judgments based on pre-construction experiments were highly predictive of judgments obtained from as-built projects (*r* = 0.86) [14] (p. 282).

### 1.2. Part 2 of a Cochrane Method

Another possible problem with using science to guide policy is that each single experiment will necessarily be limited in its scope and, inevitably, have flaws. Scientists know full well that no study is either complete or perfect. However, if findings from multiple experiments can be combined, then the applicable scope of the findings will be increased, and those pesky flaws will tend to cancel each other out (this is reader-friendly language for the theory of least squares and Fisher’s insight that randomization is the physical implementation of that theory). Cochrane solved the problem of combining findings from multiple sources with the technique of meta-analysis. Meta-analysis is the second part of the Cochrane solution.

#### 1.2.1. Meta-Analysis

Meta-analysis is used very widely. A search on the key words “meta-analysis” in the *Science Citation Index* during December, 2012, generated over 54,000 references. A general introduction to meta-analysis is given in Borenstein [26]. The use of meta-analyses to test theories is described in Cook *et al.* [27]. Mathematical implementations are given in Hartung, Knapp and Sinha [28]. The mathematics used in this article are given in Hedges and Olkin [29], because they provide a very simple model that requires only two inputs from each study: a measure of effectiveness, such as a correlation (*r*), and the *n* over which that correlation was calculated.

The connection between experimental design, meta-analysis and quality control in medicine is this: if there were a database of meta-analyses of various medical treatments for various diseases, then that database could assist medical decision-makers in selecting the best treatment for a given disease. For example, doctors now have a comprehensive database available on which drugs are most efficacious for various diseases. They can rely on the database, because all inputs must pass through the rigorous testing of randomly controlled trials. This database allows doctors to make judgments based on far more experience than any one doctor could acquire individually.

Such a database has, in fact, been implemented. The implementation has been done by the Cochrane Collaboration. Thus, the overall approach of the Cochrane Collaboration is as follows:

“How do you know if one treatment will work better than another, or if it will do more harm than good? Cochrane Reviews are systematic reviews of primary research in human health care and health policy, and are internationally recognized as the highest standard in evidence-based health care. They investigate the effects of interventions for prevention, treatment and rehabilitation. They also assess the accuracy of a diagnostic test for a given condition in a specific patient group and setting. They are published online in The Cochrane Library”.

[30]

In other words, the Cochrane approach puts the evidence into “evidence-based decision making” [31]. To date, this approach has been applied to over 5,000 medical interventions [32]. Details on the Cochrane method are given by the Cochrane Collaboration [33]. The connection between the Cochrane Collaboration and quality control in urban planning is this: if there were a database on how effective various urban design guidelines are, then planners could use that information as a quality control technique to assist in selecting the best guidelines for implementation in their own jurisdictions.

#### 1.2.2. Converting Meta-Analytic Findings into Implementation Recommendations

Table 1 lists meta-analytic findings that would be obtained by reviewing scientific data on two hypothetical alternatives: Plan A and Plan B. In this simple example, there are data from three experiments for each of Plan A and B. Altogether there are 145 environments (the number of stimuli or “nstim”) for each plan. The efficacies, or estimates of the measure of performance, for the individual experiments in Plan A were *r* = 0.85, *r* = 0.70, and *r* = 0.77. Without meta-analysis, the best we could conclude is that three experiments found strong support for Plan A. With meta-analysis, we can go further and conclude that, when combined, the collective estimate of the efficacy of Plan A is *r* = 0.76, and the probability of this results being due to random chance is very much less than 0.05. The policy recommendation would be that control would have a positive effect. The same conclusion can be reached by inspecting the 0.05 confidence interval (0.05 CI). If this interval includes 0.0, then *p* > 0.05. If this interval does not include 0.0, then *p* < 0.05. The reason for reporting both the *p*-level and the 0.05 CI is that most behavioral research uses the “*p* < 0.05” criterion, but the 0.05 CI is much more informative. The same finding can have a *p*-level <0.05, but with very narrow (well-defined) precision or with very wide (poorly-defined) precision. Reporting both measures enables readers using either criterion to interpret the findings.

The support for Plan B is different. Here, the overall efficacy is *r* = 0.13 and the *p*-level is greater than 0.05. For Plan B, the simplest scientific recommendation would be that, given the current scientific data, plan B has no discernible benefit, so the implementation would not be recommended.

Many variations on this simple meta-analysis are possible. The efficacies of two or more alternatives (e.g., Plan A *vs.* Plan B) can be compared to find out which is more efficacious. In this example, Plan A at *r* = 0.76 just plain works better than Plan B at *r* = 0.13. Experiments need not have a single factor: combinations and interrelations of multiple factors can also be analyzed; or, if there are different venues in the experiments (such as Experiments 1 and 4 showing residential streets, Experiments 2 and 5 showing shopping malls and Experiments 3 and 6 showing urban parks), then the findings reported above would indicate that, whatever it is, the efficacy of Plan A obtains over a variety of venues. Details on how to do these more complicated variations are given in Hedges and Olkin [29].

**Table 1:** Hypothetical efficacies (measured as *r*) of Plans A and B.

Individual				Collective Results			
Alternative	Experiment	nstim	*r*	Σ nstim	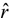	*p*-level	0.05 CI
Plan A	Exp. 1	20	0.85				
Plan A	Exp. 2	50	0.70				
Plan A	Exp. 3	75	0.77				
Synthesis	Exp. 1, 2 and 3			145	0.76	5e−28	0.68, 0.82
Plan B	Exp. 4	25	0.10				
Plan B	Exp. 5	50	0.15				
Plan B	Exp. 6	70	0.12				
Synthesis	Exp. 4, 5 and 6			145	0.13	0.075	−0.03, 0.28

## 2. Applications

The preceding sections of this article have attempted to suggest that, in theory, the Cochrane method can be applied to issues of contextual fit. This section continues the presentation by reporting three experiments that investigated the effects on the visual appeal of the three factors chosen for inquiry in this article: trees, cars or diversity.

### 2.1. Little Boxes

#### 2.1.1. The Venue

**Figure 2:**
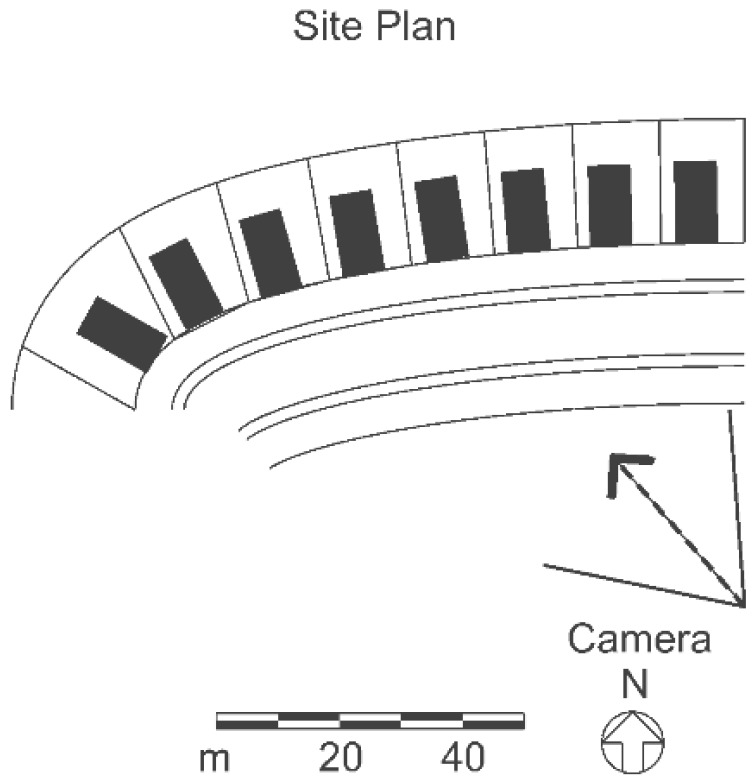
Site plan for the Little Boxes experiment.

The venue for this study was Daly City, the homogeneity of which was made famous by the folk song “Little Boxes” [34]. The scenes were blocks of eight houses. In order to eliminate possible selection bias, eight different houses were selected randomly from a book on the architecture of Daly City [35]. Each house was then modeled in a CAD program. The CAD models were used, because the afore-referenced data on simulation efficacy indicated that these models can be used to represent real environments. Colors were taken from Watch and Hope [36] (pp. 2–5) and assigned at random to the houses in each scene. Figure 2 shows the site plan.

#### 2.1.2. Experimental Design and Stimuli

**Figure 3:**
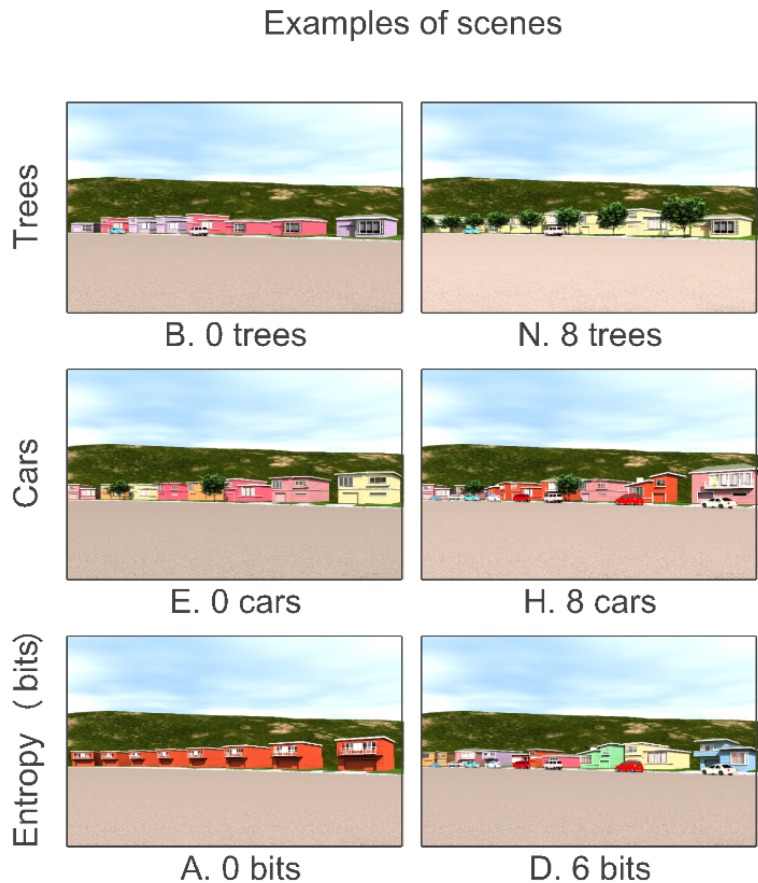
Design features and ranges of options for the Little Boxes experiment.

The experimental design was a Greco-Latin square [37] (p. 146, Plan 4.2). There was a total of 16 scenes. There were four factors: trees, cars, diversity of building shape and diversity of building color. Ranges were 0, 2, 4 and 8 for the numbers of trees and cars. The blocks in this experiment were created with 0, 1, 2 or 3 bits of shape and color entropy. Entropy has been shown to be a very strong predictor of perceived diversity [38,39,40]. For total entropy (both shape and color), the range was 0 to 6 bits. Figure 3 shows examples of the design features and the minimum and maximum option for each feature.

#### 2.1.3. Data Acquisition

The participants were 29 undergraduate engineering students, with a mean age of 20.1 years (SD = 4.2). Twenty-six were male and three were female. Political affiliations were 11 liberal, 14 moderate, two conservative and two who did not state political affiliation. Scenes were shown in a Power Point presentation during a class. Instructions were “Please look at some street scenes and rate each on a scale of not appealing (1) to appealing (8). The first two scenes show the range of variation. Please do not rate the first two scenes”. The two warm-up scenes were shown, followed by the 16 scenes in the experimental design.

#### 2.1.4. Results

**Figure 4:**
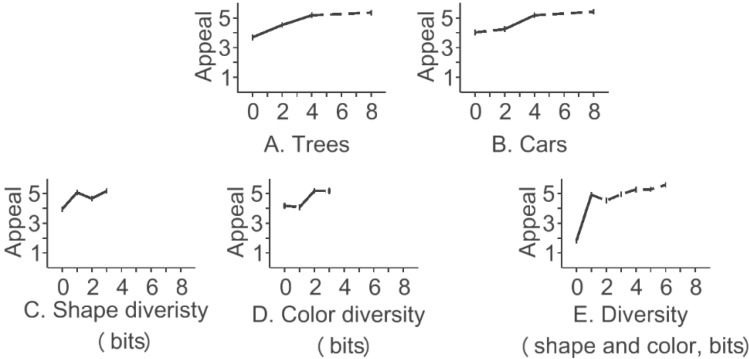
Results for the Little Boxes experiment. Solid lines indicate contrasts that achieved the individual “*p* < 0.05” level of significance.

The presence of trees had the strongest effect on visual appeal (9% of variance F(1,420) = 75.65, α = 1e−16), followed by cars (8.2%, F(1, 420) = 69.08, α = 1e−15), diversity of color (7.2%, F(1, 420) = 61.30, α = 4e−14) and diversity of shape (2.5%, F(1, 420) = 21.06, α = 1e−10). Mean values of visual appeal are shown in Figure 4. The means for trees were 3.69, 4.52, 5.28 and 5.35 for options of 0, 2, 4 and 8 trees. Inserting two trees raised the visual appeal of the block (F(1,420) = 18.99, α < 0.001), as did increasing the number of trees from 2 to 4 (F(1,420) = 15.63, α < 0.001). Increasing the number of trees from 4 to 8 did not produce a detectable difference in visual appeal (F(1,420) = 0.13, α > 0.05). For cars, there was no change in visual appeal from no cars to two cars (F(1,420) = 1.26, α > 0.05), but going from 2 to 4 cars did increase the visual appeal of the block (F(1.420) = 23.54, α < 0.001). Increasing the number of cars from 4 to 8 had no detectable effect (F(1,420) = 1.58, α > 0.05). Mean responses for shape entropy were 3.98, 5.06, 4.65 and 5.16 for options of 0, 1, 2 and 3 bits. Contrasts between options were all significant using the α < 0.05 criterion. For color entropy, the means were 3.98, 5.06, 4.65 and 5.16. Only the difference between the options of 1 and 2 bits was significant (F(1,420) = 27.91, α < 0.001). For the entropy of both shape and color, the means were 1.83, 4.91, 4.51, 4.49, 5.26 and 5.27 for options of 0, 1, 2, 3, 4, 5 and 6 bits. A significant difference was found between zero entropy (total homogeneity) and one bit (half and half), at F(1,420) = 86.24, α < 0.001.

In terms of correlations with visual appeal over *n* = *nstim* = 16, the effects were *r* = 0.50 for trees, *r* = 0.46 for cars, *r* = 0.29 for shape diversity, *r* = 0.46 for color diversity and *r* = 0.68 for diversity of color and shape.

### 2.2. Car *vs*. No Car

#### 2.2.1. The Venue

**Figure 5:**
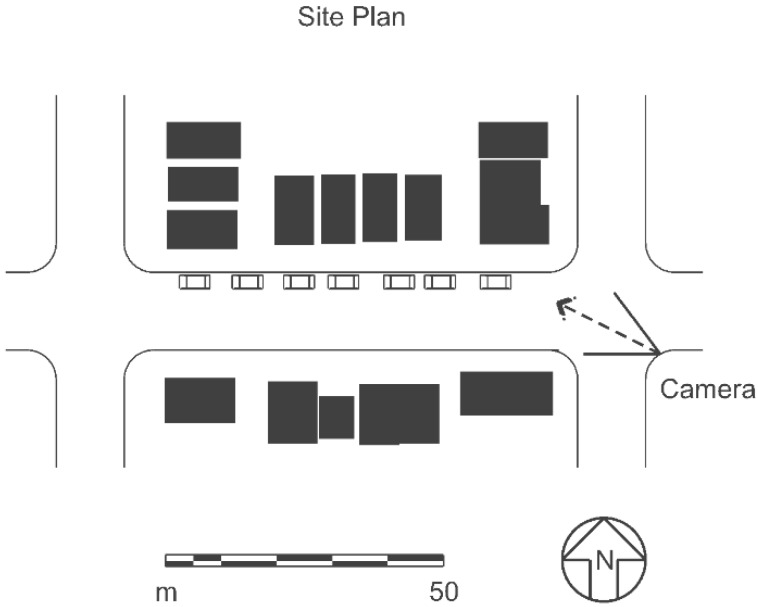
Site plan for car *vs*. no car.

There were three constraints for selecting the venue in this experiment. The first constraint was that the style of the existing houses had to be homogeneous. The reason for this was that architectural style has such a large effect on visual appeal that it has to be controlled to investigate the effects of other design features. Second, a real venue was desirable to compensate for any effects due to simulations. Third, that real venue had to have blocks of houses with and without cars. The Sunset neighborhood of San Francisco met these constraints. Most of the neighborhood was built by a single developer at one time [41], resulting in perhaps the stylistically most homogeneous neighborhood in San Francisco. In fact, it is much more uniform than the Little Boxes of Daly City, even though both venues were built by the same developer. The neighborhood also had street-cleaning days, during which, in theory, there would be no cars on the street. Figure 5 shows the site plan.

#### 2.2.2. Experimental Design and Stimuli

**Figure 6:**
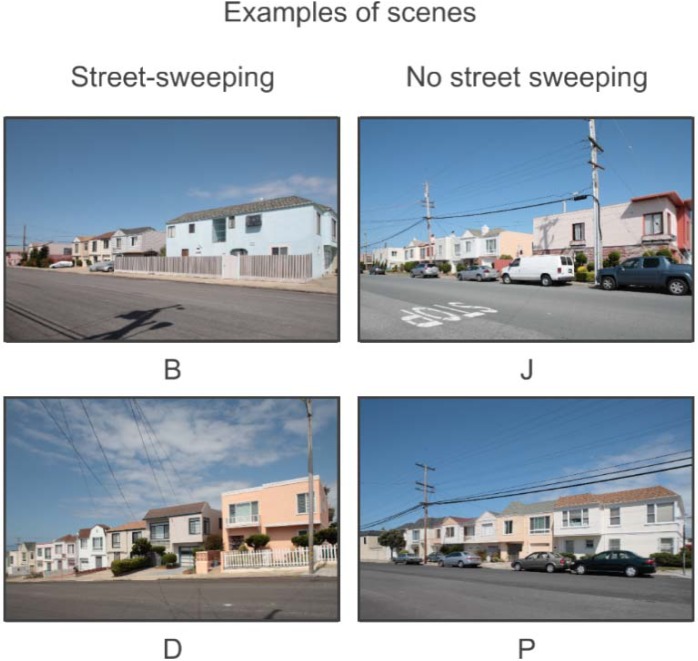
Design features and ranges of options for car *vs*. no car.

The original experimental design called for eight blocks, photographed during regular days and then again during street-cleaning days. However, after three months of attempts, it was impossible to find the required number of blocks in which there were eight contiguous sufficiently law-abiding households to satisfy the requirements of that experimental design. Accordingly, a random procedure was used to select the blocks. The random sampling began with a list of assessor’s block numbers. A random permutation of those block numbers generated the (random) order in which blocks would be visited. The blocks were then visited in that random order and photographed. The sample was complete when there were eight blocks with and eight blocks without cars. Thus, this experimental design controlled for block effects through randomization. The result was a sample of 16 scenes, eight of which had street parking and eight of which did not. Examples are shown in Figure 6.

#### 2.2.3. Data Acquisition

In the Little Boxes experiment, cars accounted for 8.2% of the variance, so that was the target for this experiment. Power analysis [42] indicated that 14 participants would be needed. Twenty-four participants were recruited by a professional survey research firm from the adult population of a major city in the United States. The participant sample was balanced for gender and political affiliation. The mean age was 49 years (SD = 17). Occupations ranged from student to attorney.

#### 2.2.4. Results

**Figure 7:**
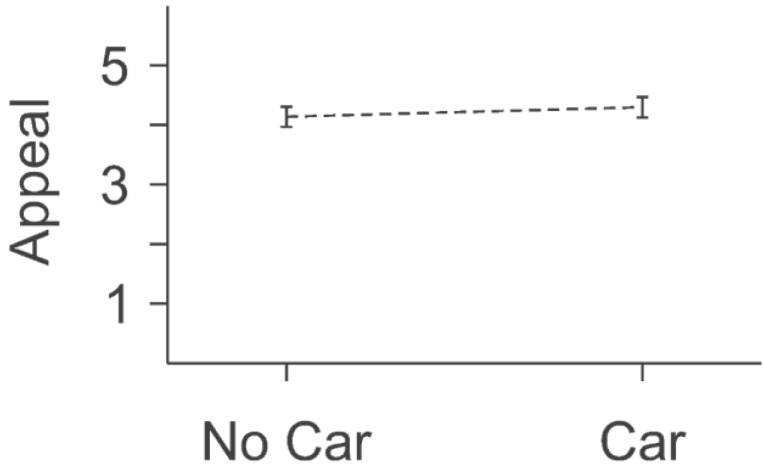
Results for car *vs*. no car. Solid lines indicate contrasts that achieved the individual “*p* < 0.05” level of significance. Dotted lines indicate contrasts that did not achieve the individual “*p* < 0.05” level of significance.

Overall, the presence of cars had a very small effect on the visual appeal of the street (0.2% of variance, F(1,345) = 1.40, α = 0.17). The contrast of cars *vs*. no cars (shown in Figure 7) repeated the overall findings (M_car_ = 4.30, M_no_car_ = 4.14, F(1,345) = 1.40, α = 0.17), to the effect that cars had a very small effect on the visual appeal of these streets. However, the results also hinted at the more detailed information that the visual appeal of streets was greater if there were cars. Given the ambiguity of this finding and previous findings for cars, it seemed appropriate to attempt replication in yet another venue.

### 2.3. “New” Amsterdam

The last experiment in this article revisited the factors of trees, car and diversity. The reason for this selection of factors was that the previous work produced ambiguous or discrepant findings, suggesting that additional work in a different venue would be useful in deciding how well the previous data generalized. For trees, the Little Boxes finding was that trees increased visual appeal. For cars, the finding from the Little Boxes was that cars increased the visual appeal of a street, while the finding of cars *vs*. no cars was that adding cars had an undetectable effect on the visual appeal of a street. For diversity, the finding was that the entropy of color had more influence on appeal than the entropy of shape, but that finding was based on a venue chosen to be highly homogeneous, so that result might or might not hold up in more complex venues.

#### 2.3.1. The Venue

**Figure 8:**
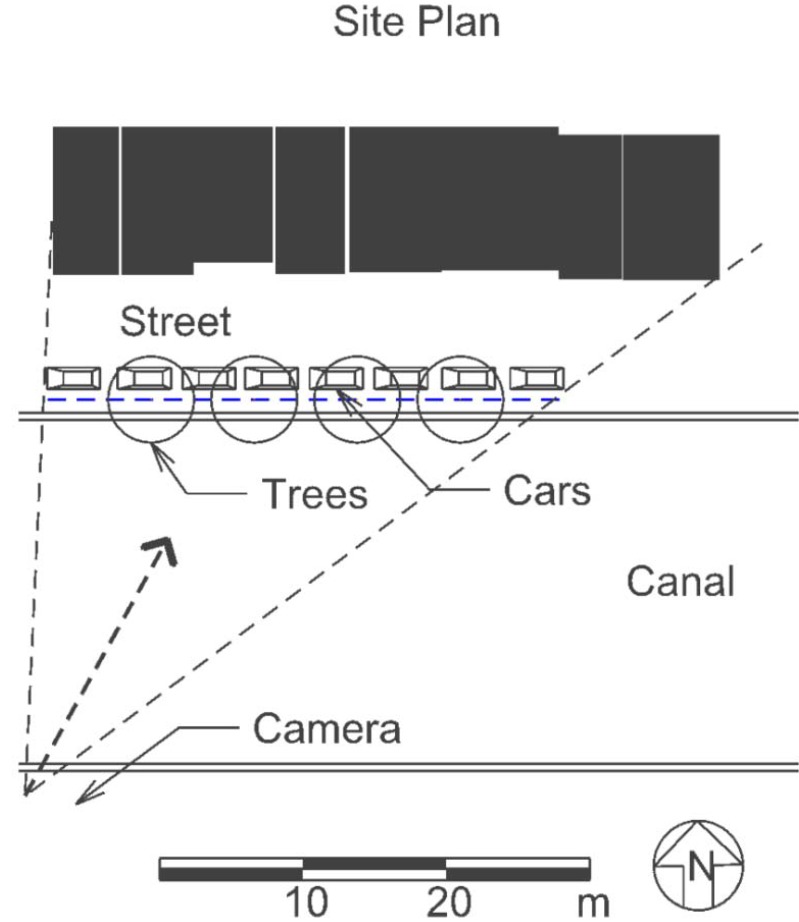
Site plan for “New” Amsterdam.

In order to enhance the data on these three factors, another experiment was done in a more complex venue that was altered to express different amounts of trees, cars and diversity. A suitable venue was Amsterdam. Accordingly, this experiment used the same design features as were used in the Little Boxes study, but the options were changed. The venue was changed from suburban America to a dense European city (Amsterdam). Houses were selected from a visit to Amsterdam, and blocks were created in a CAD program to find out how strongly trees, cars and diversity in façade color would change the visual appeal of “New” Amsterdam. Figure 8 shows the site plan.

#### 2.3.2. Experimental Design and Stimuli

**Figure 9:**
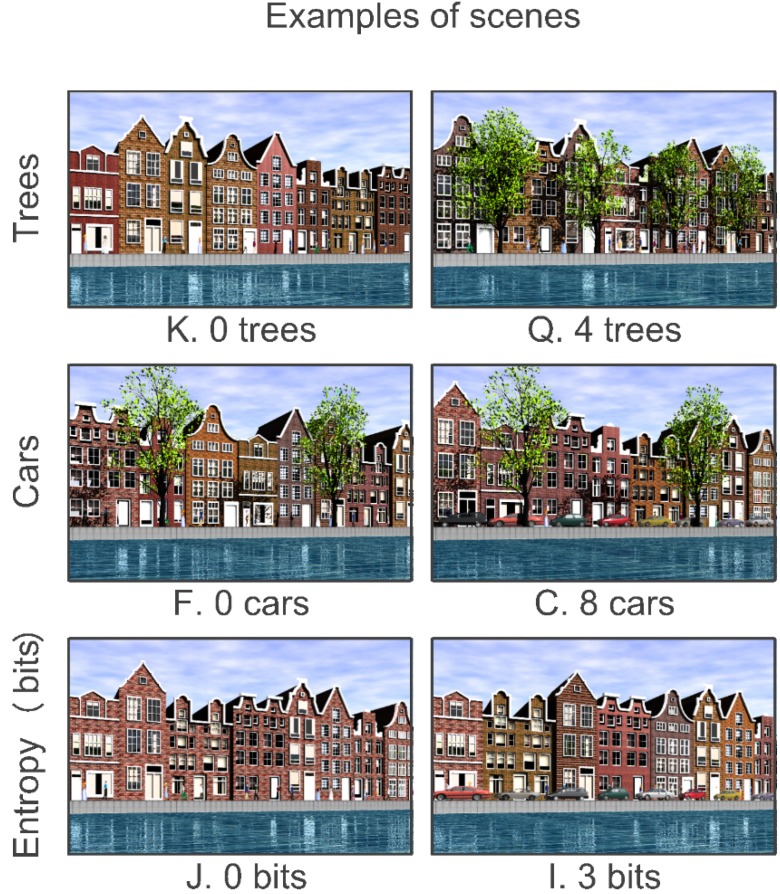
Design features and options for “New” Amsterdam.

Options for trees were 0, 2 or 4. There were either no cars or eight cars. Diversity was expressed as differences in brick color, with a range of 0, 2 or 3 bits. The CAD protocols were the same as were used in the Little Boxes study. The experimental design was a factorial of trees (3) by cars (2) by brick entropy (3), for a total of 18 scenes. Examples of the design features and options are shown in Figure 9.

#### 2.3.3. Data Acquisition

Power analysis indicated that 19 participants would be required. Twenty-four participants were recruited by a professional survey research firm. There were equal numbers of men and women and also equal numbers of political liberals, moderates and conservatives. The mean and standard deviation of age were M = 45.1 and SD = 13.5 years. Occupations ranged from social worker to business owner.

#### 2.3.4. Results

**Figure 10:**
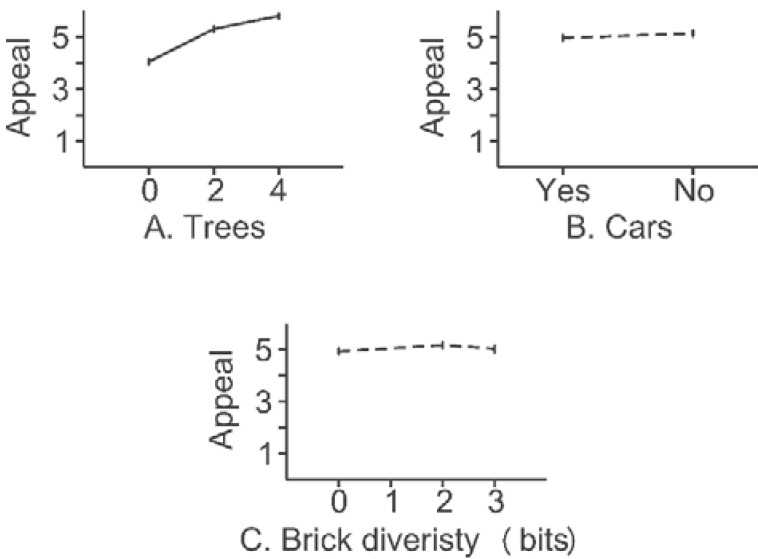
Results for “New” Amsterdam. Solid lines indicate contrasts that achieved the individual “*p* < 0.05” level of significance. Dotted lines indicate contrasts that did not achieve the individual “*p* < 0.05” level of significance.

Overall, trees dominated the responses at 12.9% of variance (F(1,391) = 155.42, α = 3e−30). Contrasts between means for each option for each feature are shown in Figure 10. Cars had an effect two orders of magnitude smaller than the tree effect (0.2% of variance, F(1,391) = 2.33, α = 0.08). The entropy of brick color was another order of magnitude smaller (0.08%, F(1,391) = 1.04, α = 0.24). For trees, the means were 4.04, 5.30 and 5.80 for options of 0, 2 or 4 trees. Increasing the number of trees increased visual appeal (F(1,391) = 72.62, α < 0.001, for increasing from 0 to 2 trees, and F(1,391) = 12.60, α < 0.001, for increasing from 2 to 4 trees). The difference between no cars and eight cars was not detectable (F(1,391) = 2.34, α > 0.05). The differences in the diversity of bricks were not detectable either (F(1,391) = 2.97, α >0.05 for *H* = 0 *versus* *H* = 2 bits; F(1,391) = 0.97, α > 0.05 for *H* = 2 *versus* *H* = 3 bits). Means are shown in Figure 10.

In terms of correlations with visual appeal of the street, the findings were *r* = 0.92 for trees, *r* = −0.11 for cars and *r* = 0.075 for the entropy of bricks, all on *n* = 18 stimuli.

## 3. Synthesis

As mentioned above, the data described in this article are not the only scientific work done on principles of contextual design. Table 1 in the Appendix describes 55 such findings, obtained from 638 scenes and 1,130 participants, on how well seven contextual fit policies would, if implemented, work. The caption in Table 1 also describes how to interpret the symbols “Experiments” “Σnstim”, “
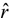
”, and “0.05 CI”. The data listed in Table 1 are useful for detailed applications and research; however, the sheer quantity of information can be over-whelming, and valid generalizations can be obscured by all of that detail. Fortunately, this is precisely the problem that can be solved with meta-analysis. Application of meta-analysis to the data in Table 1 produces the list of the efficacies of seven contextual design principles listed in Table 2.

The relevance of Table 2 to planning policy is as follows. When evaluating how features of new projects will change the visual appeal of the physical context of those projects, the most important feature so far is architectural style. Based on 191 scenes, the overall effect is *r* = 0.64. Based on the detail provided in Table A1, it seems that whether a new project does or does not match the styles of its context makes a difference. The recommendation for planning policy is, consequently, that it will be efficacious to include style as a design feature worth regulating.

**Table 2:** Efficacies of design features on street appeal.

Design Feature	Experiments	Σ nstim	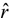	*p*-level	0.05 CI
Infill Style	29–38	191	0.64	7e−23	0.54, 0.71
Trees	46–55	252	0.62	1e−27	0.53, 0.69
Diversity	10–12, 15, 21–24, 27, 28	136	0.51	5e−10	0.37, 0.62
Infill Height	39–42	106	−0.38		−0.53, −0.20
Distance *	6–9	97	0.12	0.12	−0.08, 0.31
Third Story Setback	43–45	56	−0.10	0.23	−0.35, 0.16
Cars	1–5	106	−0.07	0.71	−0.25, 0.12

* “Distance” refers to the difference in location between the station point from which the scene was viewed and the target building. Distance was measured in meters.

For diversity, the current database consists of 136 scenes and a finding of *r* = 0.51 for the effect of diversity on visual appeal of a streetscape. Based on the detail listed in Table 1, it seems that streetscapes with more visual diversity are more appealing than streetscapes with less visual diversity. Accordingly, visual diversity is a design feature that is worth the effort needed for regulation.

The third feature is the height of new projects. This time, the current database consists of 136 scenes, and again, the relationship between infill height and the visual appeal of a streetscape is solid. Based on the detail in Table A1, it seems that when the height of an infill project is more than twice the height of the buildings in the project’s visual context, the appeal of the streetscape decreases. However, the size of the effect of infill height on the visual appeal of the streetscape (*r* = −0.38) is less than the effects of style or diversity. This indicates that, when selecting design features for regulation, a cost/benefit analysis would be helpful.

The next three design factors (distance, third story setback and cars) did not have detectable effects (*p* > 0.05). That means that there is not yet sufficient data to provide scientific justification for regulation of these factors. Thus, the current scientific recommendation for the practice of these three factors is to withhold implementation.

## 4. Discussion

This article attempted to bring together practice and research by applying the Cochrane method to issues of contextual fit. The Cochrane method, like all methods of empirical science, has its limits. However, where there are limits, there are also opportunities for further dialog. For example, in this article, choices had to be made regarding scope, technical details and whether the scientific findings should be advisory or mandatory. Of course, more than one perspective is possible. This section describes some of those possibilities and suggests keywords that may reward additional discourse.

### 4.1. Scope

As was noted above, this article focuses on the evaluation of the efficacies of specific contextual design principles. This focus allowed the application of the most basic type of experimental evidence to a planning decision: ascertaining how well one end could be achieved by alternate means. Actual planning decisions, of course, will typically involve more than one end. For example, other possible ends for planning might include transport, energy, environment, accessibility, social justice, participation, participatory planning, sustainability, urban design or affordable housing. The scientific literature contains many meta-analyses that may be useful for planners who need to incorporate these ends into their plans. For instance, a search in the *Science Citation Index* during October, 2014, on the keyword “meta-analysis” and each of the planning ends listed above located twelve possibly relevant meta-analyses [43,44,45,46,47,48,49,50,51,52,53,54]. Whether an existing meta-analysis, or, for that matter, any other type of scientific study, meets the criteria for generating valid scientific policy guidance will have to be determined on a case-by-case basis. Guidelines for making such determinations are given in [55]. Likewise, planning decisions typically also require making trade-offs among different ends. Techniques for making those trade-offs are described under the concept “quality control” [56,57,58,59,60,61,62,63,64,65].

### 4.2. Technical Details

Besides being based on empirical data, the work in this article is based on the mathematics of contemporary statistical analysis. In particular, the Cochrane method is based on the mathematics of meta-analysis. The presentation of meta-analysis provided in this article is very much a bare-bones model. The bare-bones model is adequate for addressing simple, focused questions, but more elaborate questions will require more elaborate types of meta-analyses. Guidance for performing more elaborate meta-analyses can be found in [16,26,27,28,29,66,67,68,69,70,71]. For example, there may be concern about whether an individual finding generalizes over different groups of subjects or different physical environments. In meta-analysis, this type of question is handled by considering the heterogeneity of a meta-analytic result. The details on how to analyze heterogeneity are beyond the scope of this article, but the information needed to analyze heterogeneity can be found in, among other sources [29] (pp. 108–191).

### 4.3. Advisory or Mandatory

The synthesis section of this article proposes that contextual fit guidelines should be implemented only if those guidelines are scientifically valid. This proposal raises another issue that may be of interest to planners: whether scientific findings should be used as advice or as mandatory standards. In medicine, the Cochrane library is used as an advisory service for doctors by providing the best available data on how well various medical interventions work. This information enables doctors to access much, much more information than is available from one doctor’s experience. The Cochrane library can also be used in a regulatory capacity, such as approving or disapproving drugs. A good source for information on this point is the Food and Drug Association of the United States’ presentation on how drugs are approved.

## 5. Conclusions

Much more specific guidance can be generated from the available data, but space limitations preclude inclusion of that material in this article. Accordingly, this article concludes by only suggesting that: (1) there is a need for urban design to have a better system of validation and a method for testing urban design theories and generating alternatives; (2) the same need has been addressed and solved in the discipline of medicine using randomized experimental designs for individual studies and meta-analysis for discovering results from multiple studies; (3) the methods of randomized experimental design and meta-analysis can be applied to issues in urban design, such as contextual design; and (4) the application of randomized experimental designs and meta-analysis has produced very useful information regarding seven specific contextual design principles.

## Appendix

**Table A1:** The effect size data for contextual urban design guidelines. The dependent variable is the visual appeal of a street.

#	Design Feature	Venue	Units	Range	nsubj^t^	nstim^t^	*r*	Source
1	Cars	Urban streets	Area of cars in image	0%–12%	43	32	−0.40	Stamps and Miller [72]
2	Cars	Urban streets	Cars	Present-absent	42	24	−0.03	Stamps [73]
3	Cars	Suburban streets	Cars	0–8	29	16	0.46	Little Boxes
4	Cars	Urban streets	Cars	0–7	24	16	0.13	Car *vs*. No Car
5	Cars	European streets	Cars	0–8	24	18	−0.11	“New” Amsterdam
6	Distance	Urban streets	Meters	15–37	37	24	0.19	Stamps [24]
7	Distance	Urban streets	Meters	7.5–120	18	24	0.16	Stamps [24]
8	Distance	Urban streets	Meters	22.6–34.7	24 *	24	0.01	Stamps [24]
9	Distance	Suburban streets	Meters	31.5–245	90	25	0.11	Nasar and Stamps [74]
10	Diversity	Residential blocks	H (Colors)	0.0–2.8	57	6	0.97	Stamps [38]
11	Diversity	Residential blocks	H (size)	0.0–2.8	57 *	6	0.94	Stamps [38]
12	Diversity	Residential blocks	H (silhouette)	0.0–2.8	57 *	6	0.96	Stamps [38]
13	Diversity	Residential blocks	H (silhouette)	0.0–2.8	57 *	16	(0.28)	Stamps [38]
14	Diversity	Residential blocks	H (articulation)	0.0–2.8	57 *	16 *	(0.36)	Stamps [38]
15	Diversity	Residential blocks	H (silhouette and articulation)	0.0–5.6	57 *	16 *	0.57	Stamps [38]
16	Diversity	Barns	H (Shapes)	0.0–3.0	63	17	(0.63)	Stamps [39]
17	Diversity	Barns	H (Materials)	0.0–3.0	63 *	17 *	(0.73)	Stamps [39]
18	Diversity	Barns	H (Articulation)	0.0–2.0	63 *	17 *	(0.62)	Stamps [39]
19	Diversity	Barns	H (Openings)	0.0–2.0	63 *	17 *	(0.50)	Stamps [39]
20	Diversity	Barns	H (Opening color)	0.0–2.0	63 *	17 *	(0.51)	Stamps [39]
21	Diversity	Barns	H (overall)	0.0–12.0	63 *	17	0.84	Stamps [39]
22	Diversity	Residential block faces	H (styles)	0.0–2.0	20	15	0.31	Stamps [8]
23	Diversity	Residential block faces	H (styles)	0.94–2.15	89	18	−0.04	Stamps [8]
24	Diversity	Residential block faces	H (styles)	0.94–2.55	39	18	0.06	Stamps [8]
25	Diversity	Suburban street	H (shape)	0.0–3.0	29	16 *	(0.29)	Little Boxes
26	Diversity	Suburban street	H (color)	0.0–3.0	29 *	16 *	(0.46)	Little Boxes
27	Diversity	Suburban streets	H (shape and color)	0.0–6.0	29 *	16 *	0.68	Little Boxes
28	Diversity	European street	H (bricks)	0.0–3.0	24 *	18	0.07	“New” Amsterdam
29	Infill style	Urban streets	Styles	Victorians *vs*. Stucco Box	45	20	0.96	Stamps [75]
30	Infill style	Urban Streets	Styles	Match scale and character	45 *	8	0.57	Stamps [20]
31	Infill style	Residential block faces	Styles	Sea Side: Raspberry surprise	90 *	25 *	0.96	Nasar and Stamps [74]
32	Infill style	Residential block faces	Styles	Ranch: Sea Ranch	38	18	0.30	Nasar and Stamps [74]
33	Infill style	Residential block faces	Styles	Sea Ranch: Ranch	50	18	0.15	Nasar and Stamps [74]
34	Infill style	Residential block faces	Styles	Sea Ranch: Ranch	26	18	0.70	Nasar and Stamps [74]
35	Infill style	Residential block faces	Styles	Sea Ranch: Ranch	22	18	0.47	Nasar and Stamps [74]
36	Infill style	Residential block faces	Styles	Sea Ranch: I Beam	25	27	−0.02	Nasar and Stamps [74]
37	Infill style	Residential block faces	Styles	Moss Beach to Sea Ranch	26	15	0.65	Stamps [8]
38	Infill style	Urban streets	Styles	*Avant-garde*, I Beam	18 *	24 *	0.26	Stamps [24]
39	Infill height	Urban streets	% of existing height	100–200%	45	40	−0.20	Stamps [14](pp. 226–235)
40	Infill height	Residential block faces	% of existing height	133–400%	22 *	18	−0.67	Nasar and Stamps [74]
41	Infill height	Urban streets	% of existing height	100–150%	37 *	24 *	0.39	Stamps [24]
42	Infill height	Urban streets	% of existing height	100–200%	18 *	24 *	−0.85	Stamps [24]
43	Setback	Urban streets	Top floor setback	Yes/No	24 *	24 *	0.01	Stamps [24]
44	Setback	Urban streets	Top floor setback	Yes/No	29 *	16 *	−0.14	Stamps [24]
45	Trees	Urban streets	Trees	0–2	29 *	16	−0.25	Stamps [24]
46	Trees	Urban streets	Area of trees in image	0–17%	43 *	32 *	0.32	Stamps and Miller [72]
47	Trees	German streets	Trees	Present-absent	103	18	0.39	Weber, Schnier, and Jacobsen [76]
48	Trees	Michigan streets	Trees	Number and size	149	42	0.80	Schroeder, Buhyoff, and Cannon [77]
49	Trees	Ohio Streets	Trees	Number and size	N/A	60	0.67	Schroeder, Buhyoff, and Cannon [77]
50	Trees	Urban streets	Trees	Present or absent	42 *	24 *	0.57	Stamps [73]
51		Suburban streets	Trees	Tree diameter	61	20	0.57	Lien and Buhyoff [78]
52		Streets, industrial areas, buildings	Natural elements	Present-absent	214	6	0.71	Hernandez and Hidalgo [79]
53		Urban streets	Trees	0–2	29 *	16 *	−0.25	Stamps [24]
54		Urban streets	Trees	0–8	29 *	16 *	0.50	Little Boxes
55		Urban streets	Trees	0–4	24 *	18 *	0.92	“New” Amsterdam

* The same participants were already counted in previous experiments;^t ^“nsubj” means number of subjects. “nstim” means number of stimuli.
